# Is contrast-associated acute kidney injury (CA-AKI)associated to the type of hospital and ICU? preliminay results of the nefrocon study

**DOI:** 10.1186/2197-425X-3-S1-A51

**Published:** 2015-10-01

**Authors:** C Gomez-Gonzalez, S Mas-Font, MA Alcala-Llorente, MA Garcia, M Argüeso, M Juan, Nefrocon Investigators

**Affiliations:** Hospital Infanta Luisa, Sevilla, Spain; Hospital General de Castellon, Castellon, Spain; Fundación Jiménez Díaz, Madrid, Spain; Hospital de Sagunto, Valencia, Spain; Hospital Clinico Universitario, Valencia, Spain; Hospital General, Ciudad Real, Spain

## Objetives

To evaluate the association of the type of hospital and of Intensive Care Unit (ICU) with the incidence of CA-AKI in critically ill patients in Spanish ICUs.

## Materials and Methods

Prospective multicenter-study in 34 Spanish ICUs covering a 4-month period (from December 15th, 2012 to March 15th, 2013). Research endorsed by the Spanish Society of Intensive, Critical and Emergency Care Medicine (SEMICYUC). From 1035 initial cases, exclusion criteria of uncompleted demography data and renal depuration yielded a final total of 1012 patients.

CA-AKI is defined as an absolute increase of 0.5mg/dl or 50% relative increase of serum creatinine 48-72 hours after contrast administration. Statistical analysis applying Chi-square tests with a significance level of 0.05 (software R3.1.2 for OsX). Results expressed as number(%).

## Results

Of the 34 participating hospitals, 2.9% were of Level I, 58.8% of Level II and 38.2% of Level III. Polyvalent ICUs were 27(79.5%), medical ICUs were 5(14.7%), 1(2.9%) surgical ICU and 1(2.9%) trauma ICU. In 20(58%) hospitals, preventive strategies were not available. Such strategies were available in 10.7% of the hospitals of Level I, in 18.9% of Level II and in 41.9% of the centers of Level III (p < 0.001).

The availability of protocols was significantly higher in surgical and trauma (100%) ICUs than in polyvalent (26.8%) and medical (21.8%) units. There were also significant differences in the level of the hospitals and the performed explorations: coronary angiography was the most frequent procedure in Level-I hospitals (78.6%) and in Level-II hospitals (56.7%), and computed tomography (CT)/angio-CT in Level-III hospitals (53.7%), p < 0.001.

According to the type of ICU, CT/angio-CT was the most frequent procedure in surgical (83.9%) and trauma (100%) ICUs, coronary angiography in medical ICUs (64.1%) and similar percentages of both explorations were found in polyvalent ICUs (coronary angiography in 48.4% and CT in 44.3%), p < 0.001.

However, beside such differences, the incidence of CA-AKI was independent of these variables. The incidence was 7% in Level-I hospitals, 12.8% in Level-II hospitals and 11.6% in Level-III hospitals (p > 0.005); and in 11.5% of medical ICUs, in 19.4% of surgical ICUs, in 12.2% of polyvalent units and in 3.2% of trauma ICUs (p > 0.005).

## Conclusions

Although hospital level and the type of ICU suppose significant differences in the performed procedures, these variables are not related to the incidence of CA-AKI. The availability of preventive protocols was not related either to the development of CA-AKI.

